# TLE4 acts as a corepressor of Hes1 to inhibit inflammatory responses in macrophages

**DOI:** 10.1007/s13238-018-0554-3

**Published:** 2018-06-04

**Authors:** Xiang Zhang, Xiaoyu Li, Fei Ning, Yingli Shang, Xiaoyu Hu

**Affiliations:** 1 Institute for Immunology and School of Medicine, Tsinghua University, Beijing, 100084 China; 2 Beijing Key Laboratory for Immunological Research on Chronic Diseases, Beijing, 100084 China; 3 School of Life Sciences, Tsinghua University, Beijing, 100084 China; 4 College of Veterinary Medicine, Shandong Agricultural University, Taian, 271018 China; 5 Collaborative Innovation Center for Biotherapy, Tsinghua University, Beijing, 100084 China

**Dear Editor,**

Macrophages play a key role in maintaining homeostasis and in mounting competent immune responses. Upon stimulation, one of the key effector functions of activated macrophages is production of inflammatory cytokines including interleukin 6 (IL-6) and interleukin 12 (IL-12). IL-6 and IL-12 are important for transition from innate to adaptive immunity, and thus essential for host defense against multiple pathogens (Kishimoto, 2005; Trinchieri, 2003). However, over-production of IL-6 and IL-12 contributes to a number of human autoimmune and inflammatory diseases such as inflammatory bowel disease and rheumatoid arthritis (Neurath, 2014). Thus, the production of IL-6 and IL-12 is tightly controlled by various negative regulatory mechanisms at multiple levels targeting upstream signaling pathways, sequence-specific transcription factors, epigenetic modifications, as well as post-transcriptional steps (Lyakh et al., 2008).

Hairy and enhancer of split 1 (Hes1) is a transcription repressor that belongs to a family of basic helix-loop-helix (bHLH) DNA-binding proteins and plays critical roles in the development of multiple organs and cell types (Kobayashi and Kageyama, 2014). The structure of Hes1 is highly conserved among species, which includes bHLH domain, Orange domain and WRPW motif. Functionally, mice globally deficient in the *Hes1* gene exhibit embryonic or perinatal lethality and display multiple developmental defects, indicating a critical role for Hes1 in organism development (Kobayashi and Kageyama, 2014). Recently, accumulating evidence has suggested an emerging role of Hes1 in the immune system, including regulation of T and B cell differentiation (Shang et al., 2016b), attenuation of inflammation by suppressing chemokine production (Shang et al., 2016a) and maintenance of intestinal epithelial homeostasis (Guo et al., 2018; Ueo et al., 2012). However, the exact molecular mechanisms of Hes1-mediated regulation of immune responses remain obscure.

Transducin-like enhancer of split 4 (TLE4) is a member of the TLE family of transcription corepressors. TLE proteins are the mammalian homologs of Groucho in *Drosophila* that is involved in multiple developmental processes including sex determination and neurogenesis (Buscarlet and Stifani, 2007; Chen and Courey, 2000). TLE family proteins do not bind to DNA directly but rather interact with a variety of transcription factors such as Hes1, Runx2 and T cell factor/lymphoid enhancer binding factor (TCF/LEF) to form repressor complexes (Buscarlet and Stifani, 2007). The *Tle* gene family consists of four full length *Tle* genes in mouse and human, which encode four different proteins (TLE1-4) with similar domain structures (Chen and Courey, 2000). Given the potential redundancy imposed by multiple family members, functions of the individual mammalian TLE protein are less well understood than their single *Drosophila* counterpart. Within the TLE family, TLE1 is the most studied with implicated functions in neuronal differentiation, tumorgenesis and regulation of inflammation (Buscarlet and Stifani, 2007; Ramasamy et al., 2016), whereas the functions of TLE4 remain largely unknown, especially in the immune system.

We have previously showed that Hes1 suppressed expression of key pro-inflammatory cytokines including IL-6 and IL-12 in macrophages (Shang et al., 2016a). To further confirm these observations, we examined the gene expression of *Il6* and *Il12b* in *Mx1*-Cre (WT) and *Hes1*^fl/fl^ *Mx1*-Cre (Hes1 KO) bone marrow-derived macrophages (BMDMs) in response to lipopolysaccharide (LPS) stimulation. Deficiency of Hes1 significantly promoted induction of *Il6* and *Il12b* mRNA in BMDMs by LPS in multiple independent experiments (Fig. 1A), supporting that Hes1 indeed acted as a repressor of IL-6 and IL-12. The WRPW motif of Hes1 has been shown to interact with other proteins such as transcription corepressors to mediate gene repression (Kobayashi and Kageyama, 2014). To investigate whether WRPW motif is involved in Hes1-mediated suppression in a gain-of-function system, we assessed the effects of Hes1 on expression of *Il6* and *Il12b* by luciferase assays. Overexpressing of wild-type Hes1 inhibited luciferase activities driven by the *Il6* and *Il12b* promoters in RAW264.7 mouse macrophages upon LPS stimulation while such inhibitory capacity was compromised in a Hes1 mutant lacking the WRPW motif (Hes1ΔWRPW) (Fig. 1B), suggesting that the WRPW motif is required for Hes1-mediated suppression. Together, these data demonstrated that Hes1 suppressed Toll-like receptor (TLR)-mediated inflammatory responses via the WRPW motif that is responsible for recruitment of transcription corepressors.

**Figure 1:**
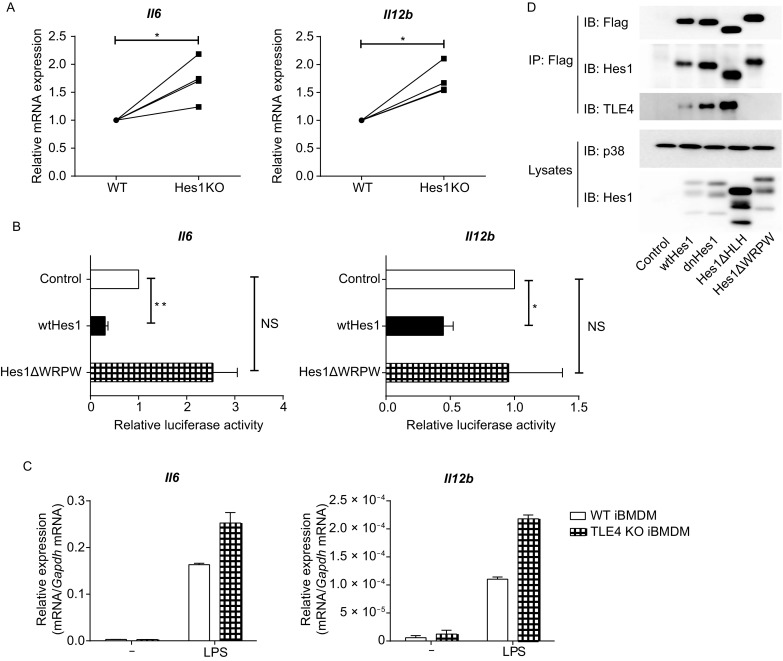
**TLE4 is required for Hes1-mediated suppression of key pro-inflammatory gene expression**. (A) Quantitative real-time PCR (qPCR) analysis of *Il6* and *Il12b* mRNA in *Mx1*-Cre (WT) and *Hes1*^fl/fl^*Mx1*-Cre (Hes1 KO) BMDMs stimulated with 10 ng/mL of LPS for 3 h, presented relative to that in WT BMDMs. (B) Luciferase activities in RAW264.7 cells co-transfected with *Il6* or *Il12b* promoter-driven reporter constructs and a Hes1 expression plasmid (pMx-Hes1, wtHes1), a Hes1 mutant expression plasmid (pMx-Hes1ΔWRPW, Hes1ΔWPRW) or control vector (pMx-GFP). 24 h post infection, cells were left untreated or stimulated with 100 ng/mL of LPS for 6 h, and cell lysates were analyzed for luciferase activities. Results are presented as ratio of luciferase activity/total protein concentration, and are normalized to values in the control vector group. (C) Quantitative real-time PCR analysis of *Il6* and *Il12b* mRNA in wild type (WT) and TLE4 KO iBMDMs stimulated with or without LPS (100 ng/mL) for 3 h. (D) Immunoblot analysis of indicated proteins in immunoprecipitated (IP) samples and whole lysates of HEK 293T cells that overexpressed wild-type Hes1 or Hes1 mutants. p38 was used as a loading control. Data are representative of three (C) independent experiments (mean ± s.d. of technical triplicates), or are pooled from four (A) or three (B) independent experiments (mean ± s.d. of biological triplicates). ns, not significant. *P < 0.05, **P < 0.01 (Student’s paired *t*-test)

Given that TLE family proteins are best characterized corepressors of Hes1 (Chen and Courey, 2000) yet there exists scarce knowledge regarding their regulation and function in the immune system, we next sought to examine the expression patterns of TLE family genes in resting and activated macrophages. Upon stimulation of macrophages with multiple TLR ligands including LPS (a TLR4 ligand), Poly(I:C) (a TLR3 ligand), zymosan (a dectin and TLR2 ligand) and R848 (a TLR7 ligand), expression of *Tle1* and *Tle2* was not significantly altered (Fig. S1A, upper panel). In contrast, expression of *Tle3* and *Tle4* was strongly induced by LPS or zymosan and modestly induced by Poly(I:C) or R848 (Fig. S1A, lower panel), indicating that TLE3 and TLE4 are likely involved in TLR-induced macrophage activation. As both TLR2 and TLR4 lead to activation of NF-κB and mitogen-activated protein kinases (MAPKs), we then investigated whether activation of NF-κB and MAPKs was responsible for TLR-mediated induction of *Tle3* and *Tle4* by pharmacologically inhibiting these signaling modules. We found that inhibition of NF-κB or MAPKs p38, JNK and ERK by specific inhibitors reduced induction of *Tle3* and *Tle4* by LPS or zymosan. Among these inhibitors, Bay11-7082 (an IKK inhibitor) and SP600125 (a JNK inhibitor) were most effective at dampening *Tle* expression (Fig. S1B). Collectively, these results demonstrated that among *Tle* genes, expression of *Tle3* and *Tle4* was upregulated upon macrophage activation in a manner dependent on the IKK and JNK signaling pathways.

Having shown that four TLE family members exhibited distinct expression patterns in macrophages, we then sought to identify the TLE family member(s) that served as Hes1 corepressor to inhibit *Il6* and *Il12b* expression. With the CRISPR/Cas9 technology, we genetically ablated each *Tle* gene in immortalized BMDMs (iBMDMs) and generated cell lines that were individually deficient in *Tle1*, *Tle2*, *Tle3* or *Tle4* (strategies for gene targeting shown in Fig. S2). Upon LPS stimulation, deficiency of TLE1, TLE2 or TLE3 did not promote *Il6* and *Il12b* expression (Fig. S3), indicating that TLE1-3 were not the corepressors of Hes1 to suppress inflammatory responses in macrophages. In contrast, ablation of *Tle4* resulted in enhanced expression of *Il6* and *Il12b* (Fig. 1C), resembling the phenotypes observed in Hes1-deficient macrophages (Fig. 1A). These results implied that functionally, TLE4 may act as the corepressor of Hes1 to regulate inflammatory responses. Given the WRPW motif of Hes1 is responsible for corepressor recruitment, we then examined whether TLE4 interacted with Hes1 through WRPW motif by co-immunoprecipitation assay. Indeed, TLE4 interacted with wild-type Hes1, a dominant-negative mutant of Hes1 (dnHes1), and a truncated Hes1 mutant lacking the HLH domain (Hes1ΔHLH), but not the truncated Hes1 mutant lacking the WRPW motif (Hes1ΔWPRW) (Fig. 1D), indicating that TLE4 physically interacted with Hes1 through the WRPW motif. Together, these functional and biochemical data demonstrated that among TLE family members, TLE4 served as the Hes1 corepressor to restrain inflammatory responses in macrophages.

To further corroborate the above observations in primary macrophages, we generated TLE4 null mice (*Tle4*^−/−^) that were globally deficient in the *Tle4* gene. Global deletion of *Tle4* led to partial lethality by 4 weeks after birth, plausibly due to growth defects and skeletal abnormalities, consistent with the previous reports (Wheat et al., 2014). Nevertheless, the surviving *Tle4*^−/−^ mice showed no observable abnormalities compared with *Tle4*^+/+^ littermates (data not shown) and thus were used for the following experiments. After confirmation of efficient deletion of *Tle4* in *Tle4*^+/−^ and *Tle4*^−/−^ cells (Fig. 2A), we generated BMDMs from *Tle4*^+/+^, *Tle4*^+/−^ and *Tle4*^−/−^ littermates and stimulated these cells with LPS. Quantitative real-time PCR analysis showed that TLE4 inhibited *Il6* and *Il12b* expression in primary macrophages in a gene-dosage dependent manner (Fig. 2B). Upregulation of *Il6* and *Il12b* upon TLE4 deficiency was highly consistent among multiple independent experiments (Fig. 2C). Moreover, protein levels of IL-6 and IL-12p40 were elevated in the supernatants of TLE4 KO BMDMs compared with those of WT BMDMs (Fig. 2D). To exclude the possibility that TLE4 may impair immune cell development, we examined populations of monocytes and lymphocytes in TLE4 KO bone marrows, and found no significant differences between WT and TLE4 KO animals (Fig. S4 and data not shown), implying a role of TLE4 in regulation of myeloid cell function instead of development. Collectively, the above genetic evidence demonstrated that TLE4 acted as a critical negative regulator of IL-6 and IL-12 in primary macrophages.

**Figure 2:**
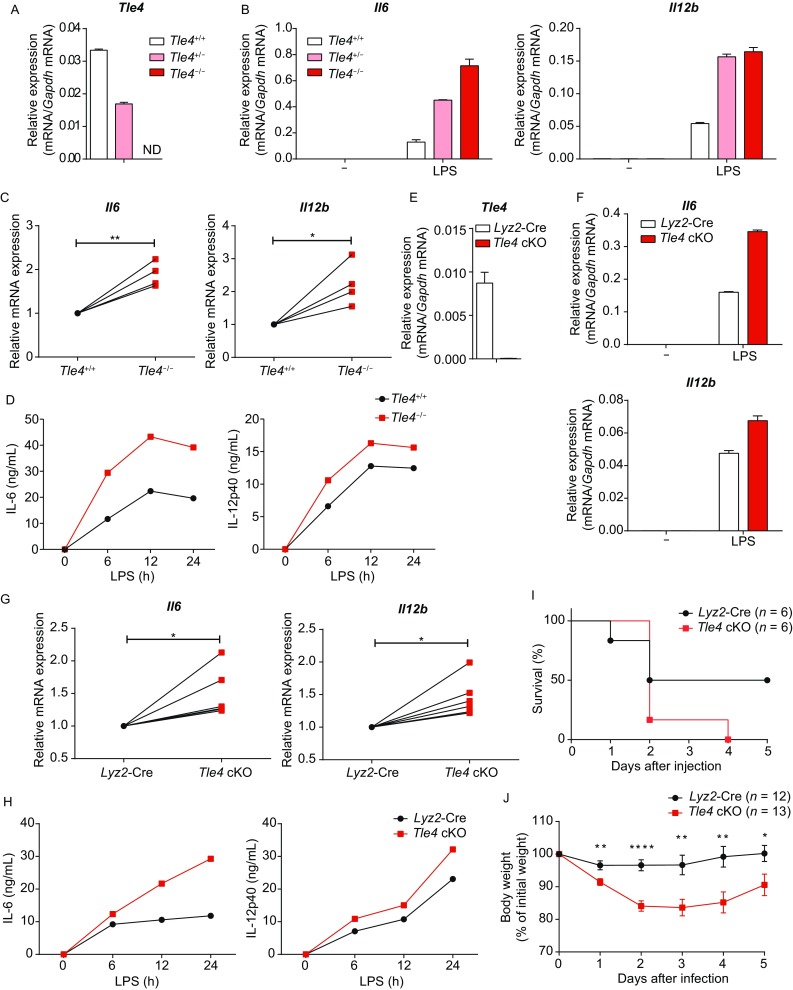
**TLE4 suppresses production of IL-6 and IL-12 and protects host from septic shock**. (A) Quantitative real-time PCR analysis of *Tle4* mRNA in BMDMs obtained from littermates of *Tle4*^+/+^, *Tle4*^+/−^ and *Tle4*^−/−^ mice. ND, not detected. (B) Quantitative real-time PCR analysis of *Il6* and *Il12b* mRNA in *Tle4*^+/+^, *Tle4*^+/−^ and *Tle4*^−/−^ BMDMs with or without LPS stimulation. (C) Cumulative results of *Il6* and *Il12b* mRNA in *Tle4*^+/+^ and *Tle4*^−/−^ BMDMs stimulated with LPS for 3 h, presented relative to that in *Tle4*^+/+^ BMDMs. (D) ELISA of IL-6 and IL-12p40 in supernatants from *Tle4*^+/+^ and *Tle4*^−/−^ BMDMs stimulated for the indicated periods with LPS. (E) Quantitative real-time PCR of *Tle4* mRNA in BMDMs from *Lyz2*-Cre and *Tle4*^fl/fl^ *Lyz2*-Cre (TLE4 cKO) mice. (F) Quantitative real-time PCR of *Il6* and *Il12b* mRNA in *Lyz2*-Cre and TLE4 cKO BMDMs with or without LPS stimulation. (G) Cumulative results of *Il6* and *Il12b* mRNA in *Lyz2*-Cre and TLE4 cKO BMDMs stimulated with LPS for 3 h, presented relative to that in *Lyz2*-Cre BMDMs. (H) ELISA of IL-6 and IL-12p40 in supernatants of BMDMs from *Lyz2*-Cre and TLE4 cKO mice stimulated for the indicated periods with LPS. (I and J) Survival rate (I) and body weight (J) of *Lyz2*-Cre and TLE4 cKO mice injected intraperitoneally with 15 mg/kg of LPS. Data are representative of four (A, B and D) or six (E, F and H) independent experiments (mean ± s.d. of technical triplicates in A, B, E and F), or are pooled from four (C), six (G), two (I), or three (J) independent experiments. *P < 0.05, **P < 0.01, ****P < 0.0001 (Student’s paired (C and G) or unpaired (J) *t*-test)

In order to specifically study the role of TLE4 in myeloid cells and to exclude the potential secondary effects from other cell types, we generated TLE4 myeloid-specific conditional knockout mice. Firstly, CRISPR/Cas9 technology was applied to knock in two loxp sites surrounding exon2 of the *Tle4* gene to obtain *Tle4*^fl/fl^ mice that were further bred with *Lyz2*-Cre mice to yield *Tle4*^fl/fl^ *Lyz2*-Cre (TLE4 cKO) mice, which specifically depleted TLE4 in myeloid cells (Fig. S5A). After confirmation of efficient deletion of *Tle4* in TLE4 cKO BMDMs (Fig. 2E), we performed flow cytometry analysis and found that the percentages of monocyte populations in bone marrows of TLE4 cKO mice were identical to those in WT animals (Fig. S5B), indicating that TLE4 deficiency did not affect myeloid cell development. Compared to the control cells, expression of IL-6 and IL-12 was increased at both mRNA and protein levels in TLE4 cKO BMDMs in response to LPS stimulation (Fig. 2F–H), further supporting the notion that TLE4 functioned as a suppressor of inflammatory responses.

Having identified that TLE4 repressed IL-6 and IL-12 expression in macrophages *in vitro*, we next sought to investigate the functions of TLE4 *in vivo* in a LPS-induced septic shock model. Upon challenge with a sub-lethal dose of LPS, TLE4 cKO mice succumbed more easily (Fig. 2I) and exhibited more severe body weight loss than *Lyz2*-Cre mice (Fig. 2J), suggesting that myeloid cell intrinsic TLE4 protected mice from LPS-induced septic shock. Thus, our results demonstrated that TLE4 suppressed expression of key immune cytokines and protected host from acute inflammation.

Production of inflammatory mediators is negatively controlled by multiple mechanisms to prevent toxicity and maintain immune homeostasis. We have previously reported that TLR stimulation induced expression of Hes1 that in turn inhibited inflammatory cytokine expression, which constituted a negative feedback loop restraining TLR-mediated inflammatory responses in macrophages (Hu et al., 2008; Shang et al., 2016b). To mediate gene repression, Hes1 recruits transcription corepressors such as TLEs via the C-terminal WPRW motif (Kobayashi and Kageyama, 2014; Chen and Courey, 2000). However, little is known about the expression patterns and functions of TLE family proteins in the immune system. In this study, we showed that expression of TLE family members TLE3 and TLE4, but not TLE1 and TLE2, was inducible in response to stimulation of macrophages with multiple TLR ligands, suggesting that TLE3 and TLE4 may play important roles in innate immune responses. Consistent with this notion, we found that TLE4 physically interacted with Hes1 and inhibited expression of IL-6 and IL-12, two key pro-inflammatory cytokines, in a pattern highly similar to inhibitory action of Hes1. Therefore, our findings identified TLE4 as a corepressor of Hes1 to restrain expression of key inflammatory genes, highlighting the importance of TLE4 in regulation of TLR-mediated inflammatory responses.

The mammalian TLE family proteins have been mainly studied in developmental processes (Chen and Courey, 2000). Although few reports have linked TLE1 with regulation of inflammatory responses (Ramasamy et al., 2016), the exact roles of TLE family members in immune cells remain largely unclear. In this study, we found that TLE4, but not the other three TLE members, inhibited expression of IL-6 and IL-12 in macrophages. According to the established paradigm, TLEs interact with Hes1 via a conserved WDR domain with little specificity (Chen and Courey, 2000). Nevertheless, our findings suggested that Hes1 specifically recruited TLE4 to repress expression of IL-6 and IL-12. One plausible explanation for such specificity may be the different expression levels of TLEs in macrophages. In fact, compared to the other three TLEs, the expression level of TLE4 was relatively high in macrophages (Fig. S1A), raising the possibility that Hes1 preferentially interacted with TLE4 to suppress inflammation in macrophages. In addition, interaction of Hes1 with different TLE proteins may lead to formation of different transcription repressive complexes by recruiting additional proteins (Buscarlet and Stifani, 2007). Further studies are needed to gain comprehensive understanding of the precise molecular mechanisms underlying Hes1-TLE interaction in the context of inflammatory responses. Moreover, TLE4 has been reported to interact with transcription factors other than Hes family proteins such as TCF/LEF involved in Wnt signaling that regulate development and functionality of multiple immune cell lineages (Staal et al., 2008). Thus, we speculate that TLE4 may also interact with TCF/LEF to regulate macrophage activation, which demands future investigation. In summary, through biochemical and genetic approaches, we uncovered a critical and previously unidentified role of TLE4 in suppressing macrophage inflammatory responses and in protecting host from acute inflammation.

## Footnotes

This research was supported by Ministry of Science and Technology of China National Key Research Projects 2015CB943200 (X. Hu), National Natural Science Foundation of China grants 31725010 (X. Hu), 81422019 (X. Hu), 81571580 (X. Hu), 91642115 (X. Hu) and 81661130161 (X. Hu), Shandong Provincial Natural Science Foundation of China grant ZR2017MC021 (Y. Shang), funds from Tsinghua-Peking Center for Life Sciences (X. Hu) and funds from Shandong “Double Top” Program 564013 (Y. Shang).

Xiang Zhang, Xiaoyu Li, Fei Ning, Yingli Shang and Xiaoyu Hu declare that they have no conflict of interest. All institutional and national guidelines for the care and use of laboratory animals were followed.

## Electronic supplementary material

Below is the link to the electronic supplementary material.

Supplementary material 1 (PDF 900 kb)
