# Supplementary material for: TLE4 acts as a corepressor of Hes1 to inhibit inflammatory responses in macrophages
Source: Protein Cell. 2018 Jun 4;10(4):300–5. doi: 10.1007/s13238-018-0554-3 (PMC6418302; doi:10.1007/s13238-018-0554-3)
Supplement: Supplementary file 1 — Supplementary material 1 (PDF 900 kb) [file 13238_2018_554_MOESM1_ESM.pdf]

# TLE4 acts as a corepressor of Hes1 to inhibit inflammatory responses in macrophages

Xiang Zhang[1,2,3], Xiaoyu Li[1,2], Fei Ning[1,2,3], Yingli Shang[4], Xiaoyu Hu[1,2,5*]

## Supplementary Materials

### Materials and methods

### Mice

C57/BL6 mice were purchased from Jackson Laboratory. *Hes1*$^{fl/fl}$ mice were originally obtained from R. Kageyama (Imayoshi et al., 2008). Mice with inducible deletion of Hes1 (*Hes1*$^{fl/fl}$*Mx1*-Cre) have been described (Shang et al., 2016). TLE4 globally deficient (*Tle4*$^{-/-}$) and *Tle4*$^{fl/fl}$ mice were generated by CRISPR/Cas9 genome editing in Beijing Biocytogen Co., Ltd. Genetic heterozygous mice (*Tle4*$^{+/-}$) were used for breeding pairs to generate *Tle4*$^{+/+}$, *Tle4*$^{+/-}$ and *Tle4*$^{-/-}$ littermates. Mice with a myeloid-specific deletion of TLE4 (*Tle4*$^{fl/fl}$*Lyz2*-Cre) were generated by crossing *Tle4*$^{fl/fl}$ animals to *Lyz2*-Cre mice. Experiments on mice were performed at 6-8 weeks of age with gender matched littermates.

### Cell Culture and Reagents

Murine BMDMs were obtained as previously described (Hu et al., 2008) and maintained in DMEM supplemented with 10% FBS and 10% supernatant of L929 cell as conditioned medium providing macrophage colony-stimulating factor (M-CSF). Cell culture grade LPS, Poly(I:C), Zymosan, and R848 were purchased from Invivogen, and were used at following concentrations unless otherwise specified: 10ng/ml (LPS), 100ng/ml (R848), 1μg/ml (Poly(I:C)), 100μg/ml (Zymosan). SP600125, SB203580,

and U0126 were purchased from Calbiochem, and Bay11-7082 was purchased from Sigma.

**Reverse Transcription and quantitative real-time PCR (qPCR)**

RNA was extracted from whole cell lysates with a Total RNA purification Kit (GeneMark) and was reversely transcribed to cDNA with a First Strand cDNA Synthesis Kit (Takara). qPCR was performed in triplicate determinants with an ABI StepOnePlus thermal cycler. Primary transcripts were measured with primers that amplify either exon-intron junctions or intronic sequences. Threshold cycle numbers were normalized to triplicate samples amplified with primers specific for glyceraldehyde-3-phosphate dehydrogenase (*Gapdh*). Primer sequences are listed as following:

*Gapdh* (forward, 5’-ATCAAGAAGGTGGTGAAGCA-3’ and reverse, 5’-AGACA ACCTGGTCCTCAGTGT-3’); *Il6* (forward, 5’-AGGCATAACGCACTAGGTTT-3’ and reverse, 5’-AGCTGGAGTCACAGAAGGAG-3’); *Il12b* (forward, 5’-AGC ACTCCCCATTCCTACTTCTCC-3’ and reverse, 5’-CACCCCTCCTCTGTCTCC TTCAT-3’); *Tle1* (forward, 5’-AATGCAGAGGCACTACGTGA-3’ and reverse, 5’-GGGCACAAATGGTGTTCAAT-3’); *Tle2* (forward, 5’- ATGGTCCCGTTCCT CACTC-3’ and reverse, 5’-CCCAGGAGGCTGTTCAGTT-3’); *Tle3* (forward, 5’-AGCTGGCTAACGAGAAGACG-3’ and reverse, 5’-CTGGGCTAGGATTGTGTT CAG-3’); *Tle4* (forward, 5’-TTCAGAATCCTGTGATCGGAT-3’ and reverse, 5’ -GCCGCTGCATCTCTGTCTT-3’); .

**Enzyme-Linked Immunosorbent Assay (ELISA)**

Cytokine secretion was quantified with IL-6 and IL-12p40 ELISA kits from BD Biosciences according to the manufacturers’ instructions.

**Generation of TLE-deficient iBMDMs**

Lentivirus vector lentiCRISPR containing the gRNAs of *Tle1*, *Tle2*, *Tle3* and *Tle4* (LentiCRISPR-TLE) were generated according to the instructions of the Zhang Lab at Harvard University (Shalem et al., 2014). HEK 293T cells were transfected with lentiCRISPR-TLE vectors together with packaging plasmids pVSVg (AddGene 8454) and psPAX2 (AddGene 12260) to make lentivirus using Fugene HD transfection reagent (Promega). iBMDMs were then infected with these lentiviruses and were selected with 2 μg/ml of puromycin (Invivogen) for 3 days. Single clone was selected by serial dilution and TLE gene deletion was confirmed by sequence.

**Co-immunoprecipitation and Immunoblotting Analysis**

pMx vectors expressing full-length Hes1 cDNA, a dominant-negative Hes1 (dnHes1), and Hes1 deleted with HLH domain or WRPW motif were generated as previously described (Shang et al., 2016). For co-immunoprecipitation, HEK 293T cells were transfected with pMx-GFP, pMx-Flag-Hes1 or pMx-Flag-Hes1 mutants using Fugene HD transfection reagent (Promega). 24 h post transfection, cells were lysed in a lysis buffer containing 10 mM Tris, 150 mM NaCl, 1% NP-40, 5 mM EDTA and 1 mM PMSF, 1 mM $NaVO_3$, and the proteinase inhibitor cocktail (Roche). Cell lysates were incubated with anti-Flag antibody (F1804, Sigma) for 4 h and then incubated with protein A/G agarose beads (Santa Cruz Biotechnology) overnight at 4ºC. Whole cell lysates were prepared as described previously (Hu et al., 2006). Whole cell lysates or immunoprecipitated extracts were then separated on 10% SDS-PAGE and transferred to PVDF membranes (Millipore) for immunoblotting with specific antibodies. Antibodies against p38 (sc-535), Hes1 (sc-25392) were purchased from Santa Cruz Biotechnology, Anti-TLE4 antibody (ab64833) was purchased from Abcam. Proteins were visualized with SuperSignal West Pico Chemiluminescent Substrate (Thermo Scientific).

**Luciferase Reporter Assay**

Murine *Il6* luciferase reporter plasmid containing a 1300 base pairs promoter sequences was generated as described previously (Hu et al., 2008). Murine *Il12b* luciferase reporter plasmid containing sequences from positions –356 to +55 was a gift from S.T. Smale (Plevy et al., 1997). RAW 264.7 cells were co-transfected in duplicate with the *Il6* or *Il12b* reporter plasmid and an expression plasmid encoding full length Hes1 (pMx-Hes1), Hes1 mutant (pMx-Hes1ΔWRPW) or a control vector (pMx-GFP) using Lipofectamine LTX reagent (Invitrogen). 24 h post transfection, cells were stimulated with LPS (100 ng/ml) for 6 h and cell lysates were prepared and analyzed using Dual-Luciferase Report Assay System (Promega). The total protein concentration of cell lysates was measured with BCA Protein Assay Kit (Thermo Scientific Pierce) and was used as an internal control.

**Flow Cytometry**

Bone marrow cells were lysed with ACK lysing buffer (Gibco) to exclude red blood cells and were stained with antibodies on ice for 30 min in the dark. APC anti-mouse Ly6G (1A8, 1:400, BD Biosciences), PE anti-mouse Ly6C (AL21, 1:400, BD Biosciences), Alexa Flour 700 anti-mouse CD45R/B220 (RA3-6B2, 1:200, Biolegend) and Pacific Blue anti-mouse CD4 (RM4-5, 1:200, Biolegend) together with PE/Cy7 anti-mouse CD11b (M1/70, 1:400, BD Biosciences) were used to stain monocytes, B cells and T cells respectively. Cells were washed three times and were analyzed on FACSAira II flow cytometer (BD Biosciences).

**Statistical Analysis**

P values were calculated with a two-tailed paired or unpaired Student's *t* test. P values of 0.05 or less were considered significant.

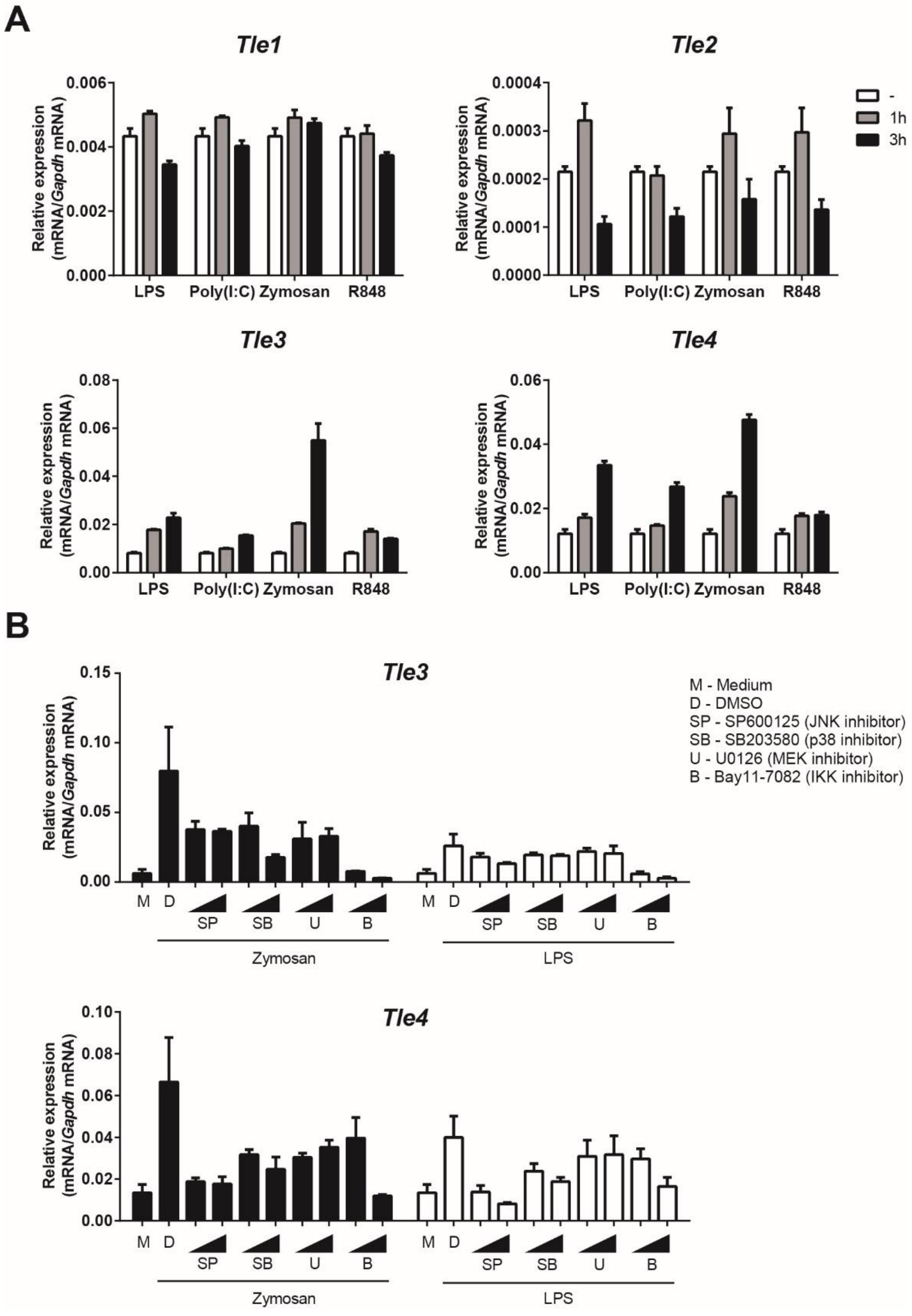


**Figure S1. Expression patterns of TLE family genes in macrophages.** (A) qPCR analysis of *Tle1*, *Tle2*, *Tle3* and *Tle4* mRNA in wild-type BMDMs stimulated with LPS, Poly(I:C), zymosan, or R848 for indicated periods. (B) qPCR analysis of *Tle3* and *Tle4* mRNA in wild-type BMDMs pre-treated with DMSO, JNK inhibitor (SP600125), p38

inhibitor (SB203580), MEK inhibitor (U0126), or IKK inhibitor (Bay11-7082) for 1h and then stimulated with LPS, or zymosan for 3h. Data are representative of two independent experiments (mean + s.d. of technical triplicates).

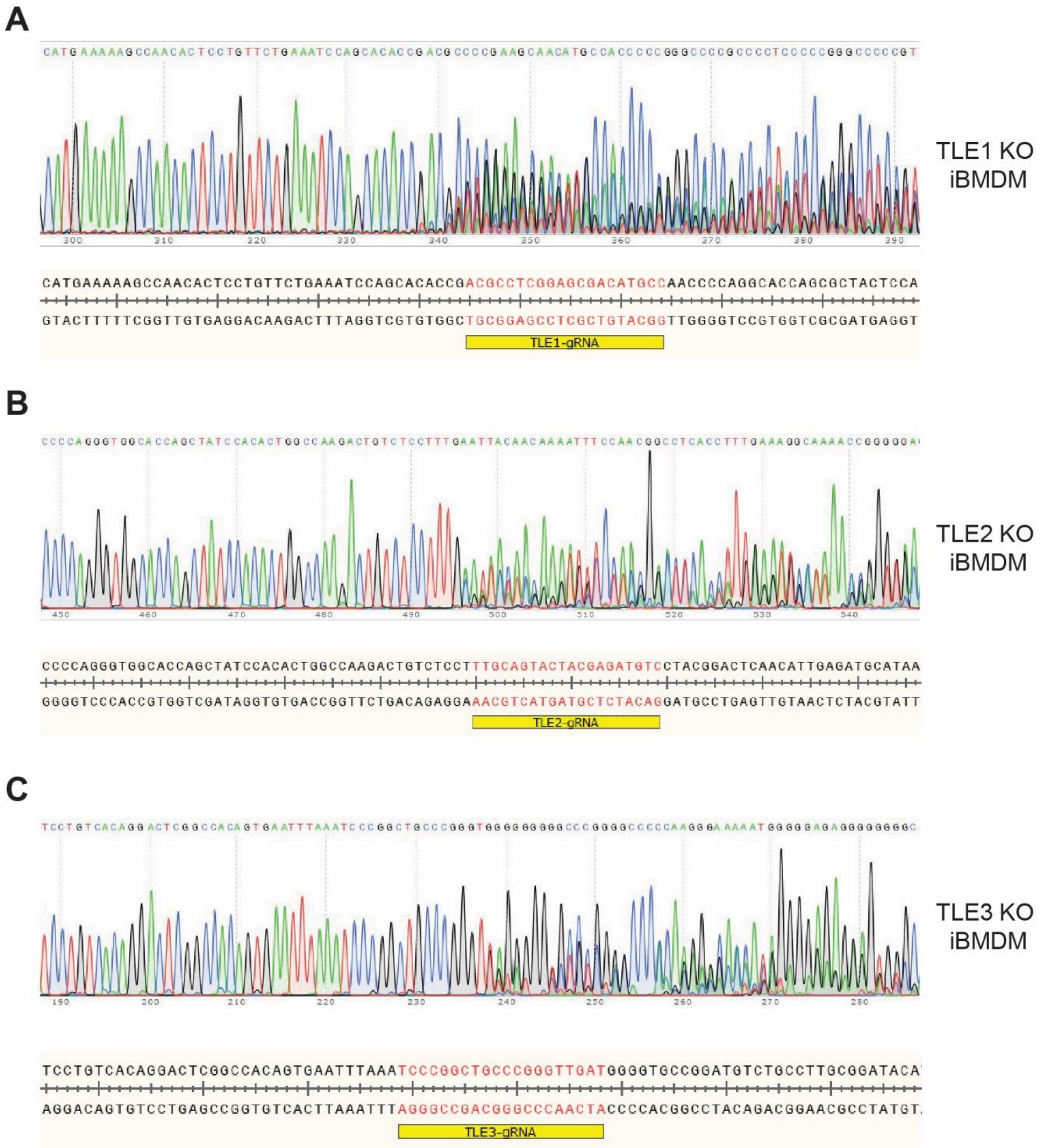


**Figure S2. TLE1-3 are effectively knocked out in iBMDMs**. DNA sequencing results of *Tle1* (A upper panel), *Tle2* (B upper panel), or *Tle3* (C upper panel) gene loci after CRISPR-Cas9-mediated gene editing in iBMDMs, and DNA sequences of unedited *Tle1* (A lower panel), *Tle2* (B lower panel), or *Tle3* (C lower panel) genes flanking gRNA targeting regions. TLE1-3 gRNAs are colored red.

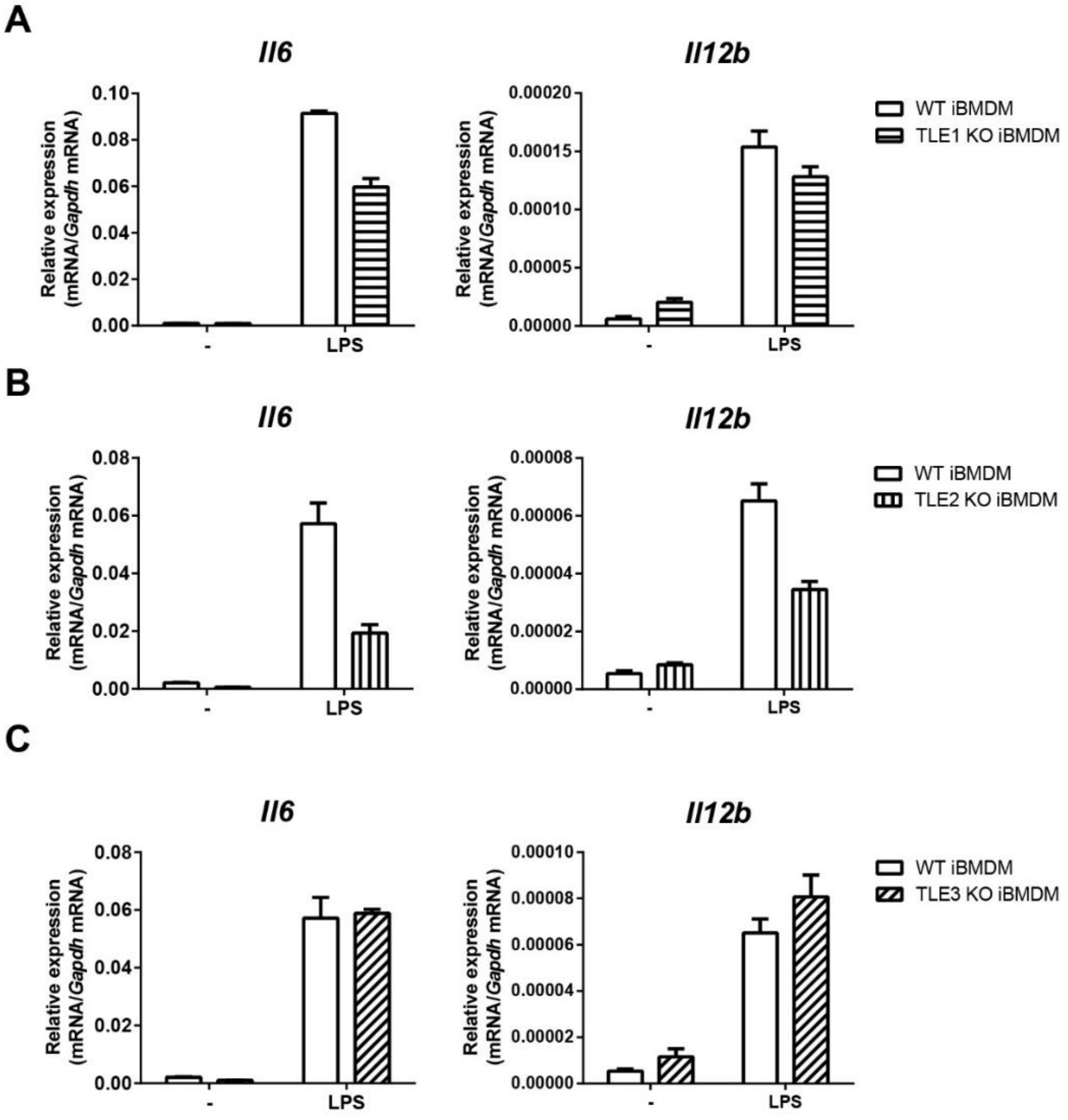


**Figure S3. TLE1-3 do not inhibit expression of *Il6* and *Il12b* in macrophages**. qPCR analysis of *Il6* and *Il12b* mRNA in wild type (WT) and TLE1 KO (A), TLE2 KO (B), or TLE3 KO (C) iBMDMs stimulated with or without LPS (100ng/ml) for 3 h. Data are representative of three independent experiments (mean + s.d. of technical triplicates).

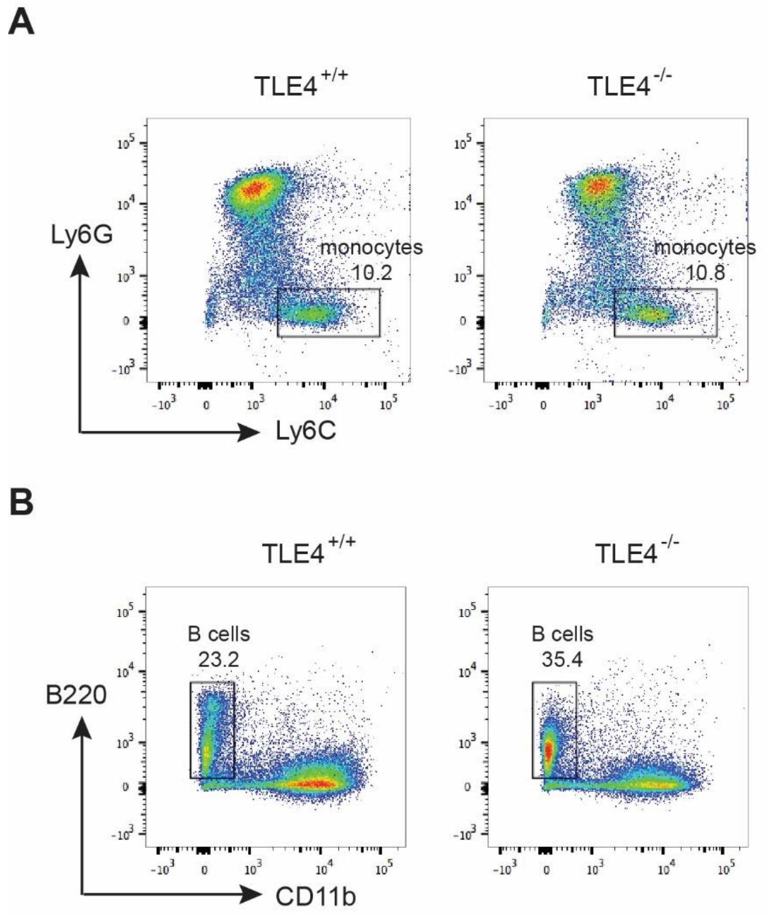


**Figure S4. TLE4 deficiency does not affect development of monocytes and B cells in bone marrows.** FACS analysis of monocytes (A) and B cells (B) in the bone marrows of $TLE4^{+/+}$ and $TLE4^{-/-}$ mice. Data are representative of two independent experiments.

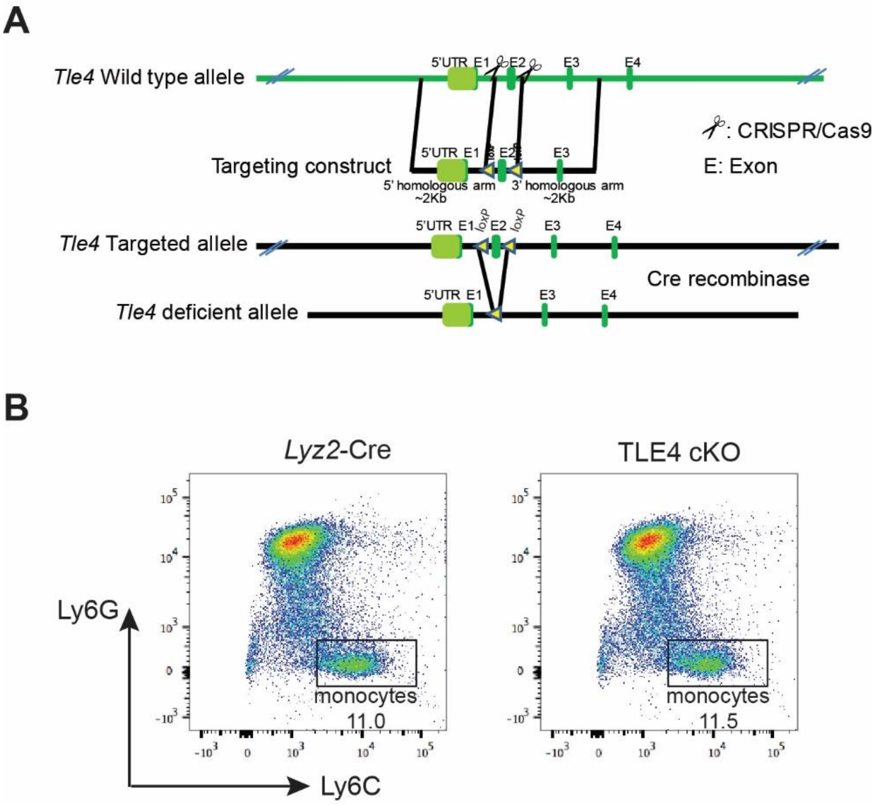


**Figure S5. Myeloid-specific depletion of TLE4 does not alter monocyte development in bone marrows.** (A) Schematic representation for generation of *Tle4* conditional KO mice. (B) FACS analysis of monocytes populations in the bone marrows of *Lyz2*-Cre and *Tle4*$^{fl/fl}$ *Lyz2*-Cre (TLE4 cKO) mice. Data are representative of two independent experiments.

## REFERENCES

Hu, X., Chung, A.Y., Wu, I., Foldi, J., Chen, J., Ji, J.D., Tateya, T., Kang, Y.J., Han, J., Gessler, M. et al (2008). Integrated regulation of Toll-like receptor responses by Notch and Interferon-γ pathways. Immunity 29, 691-703.

Hu, X., Paik, P.K., Chen, J., Yarilina, A., Kockeritz, L., Lu, T.T., Woodgett, J.R., and Ivashkiv, L.B. (2006). IFN-gamma suppresses IL-10 production and synergizes with TLR2 by regulating GSK3 and CREB/AP-1 proteins. Immunity 24, 563-574.

Imayoshi, I., Shimogori, T., Ohtsuka, T., and Kageyama, R. (2008). Hes genes and neurogenin regulate non-neural versus neural fate specification in the dorsal telencephalic midline. Development 135, 2531-2541.

Plevy, S.E., Gemberling, J.H., Hsu, S., Dorner, A.J., and Smale, S.T. (1997). Multiple control elements mediate activation of the murine and human interleukin 12 p40 promoters: evidence of functional synergy between C/EBP and Rel proteins. Mol Cell Biol 17, 4572-4588.

Shalem, O., Sanjana, N.E., Hartenian, E., Shi, X., Scott, D.A., Mikkelsen, T.S., Heckl, D., Ebert, B.L., Root, D.E., Doench, J.G. et al (2014). Genome-Scale CRISPR-Cas9 Knockout Screening in Human Cells. Science 343, 84-87.

Shang, Y., Coppo, M., He, T., Ning, F., Yu, L., Kang, L., Zhang, B., Ju, C., Qiao, Y., Zhao, B. et al (2016). The transcriptional repressor Hes1 attenuates inflammation by regulating transcription elongation. Nat Immunol 17, 930-937.
